# Phytochemical, antioxidant, enzyme activity and antifungal properties of *Satureja khuzistanica in vitro* and *in vivo* explants stimulated by some chemical elicitors

**DOI:** 10.1080/13880209.2020.1743324

**Published:** 2020-04-07

**Authors:** Farzaneh Fatemi, Mohammad Reza Abdollahi, Asghar Mirzaie-asl, Dara Dastan, Kalliope Papadopoulou

**Affiliations:** a Department of Agronomy and Plant Breeding, Faculty of Agriculture, Bu-Ali Sina University, Hamedan, Iran; b Department of Plant Biotechnology, Faculty of Agriculture, Bu-Ali Sina University, Hamedan, Iran; c Medicinal Plants and Natural Products Research Center, Hamadan University of Medical Sciences, Hamadan, Iran; d Department of Pharmacognosy and Pharmaceutical Biotechnology, School of Pharmacy, Hamadan University of Medical Sciences, Hamadan, Iran; e Department of Biochemistry and Biotechnology, University of Thessaly, Larissa, Greece

**Keywords:** Methyl jasmonate, multi-walled carbon nanotubes, nodal segments culture, rosmarinic acid, salicylic acid

## Abstract

**Context:**

*Satureja khuzistanica* Jamzad. (Lamiaceae), is known for its antifungal and antioxidant compounds, especially rosmarinic acid (RA).

**Objective:**

The study examines the effect of elicitors on RA production and phytochemical properties of *S. khuzistanica.*

**Materials and methods:**

*In vitro* plants were treated with methyl jasmonate (MeJA) and multi-walled carbon nanotubes (MWCNTs). *In vivo* plants were treated with MWCNTs and salicylic acid (SA). RA was measured by HPLC. Catalase (CAT), guaiacol peroxidase (POD) and ascorbate peroxidase (APX) were quantified. DPPH and β-carotene were assayed in *in vivo* extracts. The antifungal effects of extracts were evaluated against *Fusarium solani* K **(**FsK).

**Results:**

The highest RA contents of *in vitro* plants were 50 mg/L MeJA (140.99 mg/g DW) and 250 mg/L MWCNTs (140.49 mg/g DW). The highest *in vivo* were 24 h MWCNTs (7.13 mg/g DW) and 72 h SA (9.12 mg/g DW). The maximum POD and APX activities were at 100 mg/L MeJA (5 and 4 mg protein, respectively). CAT had the highest activities at 50 mg/L MeJA (2 mg protein). DPPH and β-carotene showed 50% and 80% inhibition, respectively. The FsK aggregation was the lowest for *in vitro* extract in number of conidia [1.82 × 10^10^], fresh weight (6.51 g) and dry weight (0.21 g) that proved RA inhibitory effects. The callus reduces FsK growth diameter to 2.75 on the 5th day.

**Discussion and conclusions:**

Application of MeJA, SA, and MWCNTSs could increase RA in *S. khuzistanica* and highlighted potential characteristics in pharmaceutical and antifungal effects.

## Introduction

Plants are important sources of medicinal components for drug development. Plant secondary metabolites are unique sources of pharmaceuticals, food additives, flavours, and other industrial uses (Rates 2001). The genus *Satureja* (Savoury) (Lamiaceae) comprises about 200 species worldwide (Momtaz and Abdollahi 2010). These are highly valuable medicinal plants, mainly distributed in the Middle East and Mediterranean regions. Fourteen species of this genus grow in northern, northwestern, western, southwestern, and central parts of Iran (Hadian et al. 2011). About 30 species of this genus are commonly named savoury, among which *Satureja khuzistanica* Jamzad. is a medicinal plant endemic to Iran (Ghorbanpour and Hadian 2015). The plants have traditionally been used in Iran for its antifungal, antibacterial, antioxidant, antidiabetic, antihyperlipidemic, antibiofilm, and anti-inflammatory compounds (Hadian et al. 2011).

There is another group of phenolic compounds in extracts of this species; the main one is rosmarinic acid (RA). That is why the use of *S. khuzistanica* has become important in recent years in medicine and pharmacology research. RA belongs to the group of polyphenols which is found most notably in many Lamiaceae plants, and has antioxidant, anti-inflammatory, and antimicrobial activities. RA helps to prevent cell damage caused by free radicals, thereby reducing the risk of cancer and atherosclerosis.

Several factors such as biotic and abiotic stresses can affect plant secondary metabolites. Application of elicitors effectively promotes the production and yield of plant secondary metabolites *in vivo* in an easy and direct manner (Xu et al. 2011). In addition, *in vitro* techniques are applied both to rescue the plant species and to produce biologically active compounds. It has been reported that *in vitro* plant cultures induce higher amounts of RA than the wild plants (Xu et al. 2011). The most important factor enhancing metabolite synthesis is the elicitation process (Xu et al. 2011). Among signalling molecules salicylic acid (SA) and multi-walled carbon nanotubes (MWCNT) have been applied as oxidative stresses to induce RA production in some species. On the other hand, jasmonic acid (JA) and its methyl ester methyl jasmonate (MeJA) have a main role in a signal transduction process. These compounds are also widely used in plant cell cultures to activate secondary metabolite and defence genes. It seems that RA acts as a main accumulated defence compound against pathogens, herbivores and can also reduce oxidative damage (Hadian et al. 2011). MWCNTSs (Ghorbanpour and Hadian 2015) and SA (Vicent and Plasencia 2011) were known as potent enhancers of some secondary metabolites in previous studies. Several studies showed that SA signalling pathway was involved in biosynthesis of terpenoids (Xu et al. 2011). Researchers demonstrated that lower concentrations of SA could increase monoterpene contents in *Houttuynia cordata* Thunb. (Saururaceae) (Xu et al. 2011).

Nanoparticles could act as signal compounds inducing metabolic and physiological responses in *Pelargonium zonale* L. L’Hér. (Geraniaceae) (Hatami and Ghorbanpour 2014). Previous research reported that nanosilver particles could cause oxidative stress, raise lipid peroxidation, and catalase activity in *Artemisia annua* L. (Compositae) (Zhang et al. 2013). Also, some studies revealed that nanoparticles could activate plant antioxidant systems and increase activities of superoxide dismotase (SOD), catalase (CAT), ascorbate peroxidase (APX), and guaiacol peroxidase (GPX) in spinach chloroplast (Lei et al. 2008). It was shown that MWCNTs had ability to enhance the growth of tobacco cells at different concentrations (Khodakovskaya et al. 2012). In this study, two separate experiments were performed. At the first experiment, different concentrations of MWCNTs and MeJA were applied on nodal segments cultures of *S. khuzistanica* grown *in vitro.* The second one was performed to investigate the potential effects of spraying 100 mg/L MWCNTs and 200 mg/L SA at different time points on *in vivo* plants. Phytochemical responses, including RA contents, enzyme activities; catalase (CAT), guaiacol peroxidase (POD) and ascorbate peroxidase (APX) were measured in both *in vitro* and *in vivo* treated plants. Antioxidant activities of the extracts (DPPH and β-carotene) were measured in *in vivo* plants exposed to MWCNTs and SA treatments at different times from initial treatments. Finally, the antifungal effects of *in vivo* and *in vitro* extracts and also inhibitory impacts of callus were evaluated.

## Materials and methods

### Plant materials and growth condition

*S. khuzistanica* (NCBI:txid1114304) (Jamzad 1994) plants were supplied from Research farm of Khorraman Pharmaceutical Co., Lorestan, Iran. The plants were grown in natural light conditions (about 450–600 μmol m^−2^ s^−1^ and 28–32 °C/18–24 °C day/night) in a greenhouse at Bu-Ali Sina University, Hamedan, Iran. Plants were watered as required and fertilised every month.

### First experiment: establishment of nodal segment culture and treatment with MWCNTs and MeJA elicitors

Establishment of nodal segment culture was done in *S. khuzistanica* species. Suitable nodal segments (segment with 1 node) were cut from *in vivo* plants grown in the greenhouse at reproductive stage and were washed under running tap water for 10 min. The nodal segments were surface sterilised by 1.5% (v/v) sodium hypochlorite for 5 min, and then rinsed three times in sterile distilled water for 5 min. Twenty sterilised nodal segments were transferred to the 250 mL Erlenmeyer flask containing 100 mL liquid MS (Murashige and Skoog 1962) culture medium supplemented with 2 mg/L 6-benzylaminopurine (BAP), 3% (w/v) sucrose and 0.2% (w/v) polyvinylpyrrolidone (PVP). The samples were placed at 24 ± 2 °C in a growth chamber with a photosynthetic photon flux density of 40 µmol m^−2^ s^−1^ (provided by fluorescent tubes) and a 16/8 h light/dark photoperiod with continuous shaking at 120 rpm. They were sub-cultured to proliferate every 2 weeks into new liquid MS media supplemented with materials as described above. The MWCNTs (Iranian Nanomaterials Pioneers Co., Mashhad, Iran) were sterilised by autoclave (121 °C and 1.2 bar) and was dispersed in distilled water using ultrasonic vibration for 30 min. Seven days after the transfer of nodal segments into the new liquid MS culture medium, the dispersed stock solution of MWCNTs was added to the nodal segment culture to obtain appropriate concentrations (0, 50, 100, 250 mg/L). Also, 7-day-old nodal segments were treated by MeJA (Merck Company). The stock solution was filter sterilised and added to nodal segment cultures at different concentrations (0, 50, 100, 250 mg/L). In both MWCNTs and MeJA elicitors, samples were taken after 48 h from elicitations, frozen in liquid nitrogen and stored at −80 °C for further usage.

### Second experiment: treatment of *in vivo* grown plants with MWCNTs and SA elicitors

In this experiment, *in vivo* greenhouse plants of *S. khuzistanica* at the full flowering stage were sprayed by 100 mg/L MWCNTs and 200 mg/L SA (Merck Company). The volume of the spray was 100 mL per plant. The untreated plants (control) were sprayed with distilled water. The treated and non-treated plants were then zipped with a vinyl pack for 1 h. The shoots from the treated and untreated (control) plants were randomly sampled at 0, 24, 48, and 72 h after treatment.

### Hydromethanolic extraction and HPLC analysis

*In vitro* and *in vivo* samples of *S. khuzistanica* were harvested and dried in a dark condition at room temperature. The samples were minified (1000 mg) and suspended in methanol/water (80/20 v/v) in 250 Erlenmeyer flasks, placed in darkness for 2 days. Then, the mixtures were kept in continuous shaking at 80 rpm for 5 h followed by sonication for 30 min. Finally, the homogenate was filtered through Whatman paper (No. 1) and the obtained supernatant was evaporated at 50 °C using rotary evaporator (Heidolph, Germany). The residues were dried and the hydroalcoholic extract was scraped and stored in a dark place. Nine different concentrations of RA standard were prepared in 1 mL methanol/water (50/50 v/v) ranging from 1 to 200 mg/L. The peak areas obtained from the injections were used to calculate the calibration curve. The HPLC column was a Spherisorb ODS-2 (5 mg/L) reversed phase 4.6 mm × 250 mm. Elution was performed at a flow rate of 1.0 mL/min at 25 °C and detection at 333 nm. The injection volume was 20 μL. Two mobile phases, A and B, were used; mobile phase A was H_2_O and mobile phase B was methanol. Solvent composition of gradient was in low Pressure, 75% A and 25% B for the first 5 min, followed by 50% A and 50% B for the next 10 min, and finally 100% B for an additional 15 min. Each extract (2 mg) was dissolved in 1 mL methanol/water (50/50 v/v) and were filtered through a 0.45 µm filter. The chromatography peak of RA was confirmed according to the retention time of the reference standard. The quantitative analysis was performed with external standardisation by measurement of the peak areas using Agilent ChemStation software.

### Antioxidant enzyme activity

*In vitro* and *in vivo* samples (200 mg) were homogenised with liquid nitrogen. Then 2 mL of phosphate buffer (pH = 7.8) was added and centrifuged at 13000 *g* for 15 min at 4 °C. The supernatant was used for assessment of catalase (CAT), guaiacol peroxidase (POD) and ascorbate peroxidase (APX) activities. CAT (EC 1.11.1.6) activity was determined by measuring the consumption of H_2_O_2_ according to the method of Aebi (1984). POD (EC 1.11.1.7) activity was measured in accordance with the method of Maehly and Chance (1954). APX (EC 1.11.1.11) activity was assayed according to the method of Nakano and Asada (1981).

### Radical-scavenging assay

In this experiment, the radical-scavenging abilities were investigated in the extracts derived from *in vivo S. khuzistanica* plants treated with MWCNTs and SA through 2,2-diphenylpicrylhydrazyl (DPPH) assay and β-carotene bleaching assay.

### DPPH assay

In this assay, the radical-scavenging abilities of the extracts were tested to determine their antioxidant activity through the bleaching of the purple-colored methanol solution of DPPH (Sigma_Aldrich, Munich, Germany) by spectro-photometer. The extract (50 μL), dissolved in methanol, was added to 5 mL of DPPH solution (0.004% DPPH in methanol). Synthetic standard of Trolox (1 mM), a stable antioxidant, was used as a reference. Also, 2 mL of the hydroalcoholic extract (50 mg/L) was added to an equal volume of DPPH solution (125 μM in 1 mL of methanol) concomitant with vitamin C (5 μg/mL) as a positive control for measuring the radical scavenging activity of the hydromethanolic extracts. After 30 min of incubation at room temperature, the absorbance was recorded at 517 nm against a blank using UV_Vis spectrophotometer. The inhibition percentage of the DPPH free radical was calculated according to the following equation (Khodakovskaya et al. 2012):

$$I\%\;=\;\left(A_{\text{blank}}–{\text{ A}}_{\text{sample}}/A_{\text{blank}}\right)\;\times \;100$$

where, A_blank_ is the absorbance of the control reagent (containing all reagents except the test compound), and A_sample_ is the absorbance of the test compound at particular times. All the assays were carried out three times.

### β-Carotene bleaching assay

The antioxidant activity of the extracts was determined using the β-carotene/linoleic acid test (Dapkevicius et al. 1998). Approximately 10 mg of β-carotene (type I synthetic) were dissolved in 10 mL of chloroform. Then, 0.2 mL of this solution was added to a boiling flask containing 20 mg linoleic acid and 200 mg Tween 40. Chloroform was removed using a rotary evaporator at 40 °C for 10 min. Then, 50 mL of oxygenated distilled water was added slowly with vigorous stirring to form an emulsion. The emulsion (5 mL) was added to a tube containing 0.2 mL of extract solution prepared according to Choi et al. (2000). The absorbance was immediately measured at 470 nm against a blank consisting of an emulsion without β-carotene. The tubes were placed in a water bath at 50 °C and the oxidation of the emulsion was monitored spectrophotometrically by measuring absorbance at 470 nm over a 60 min period. Samples containing 0.2 mL of ethanol were also monitored and used as control. The same procedure was carried out for the stable antioxidant reference BHT (butylated hydroxytoluene) as positive control and blank. The antioxidant activities of the extracts were expressed as an inhibition percentage with reference to the control sample after 60 min of incubation, using the following formula (Duarte-Almeida et al. 2006):

$$\text{AA}\;=\;100\left({\text{DR}}_{C}-{\;\text{DR}}_{S}\right){/\text{DR}}_{C}$$

where, AA = antioxidant activity; DR_C_ = degradation rate of control = [ln (a/b)/60]; DR_S_ = degradation rate of sample = [ln (a/b)/60]; a = absorbance at time 0; b = absorbance at 60 min.

### Media preparation for inhibitory effects of *in vitro* and *in vivo* extracts and calluses of *S. khzistanica* on *Fusarium* *solani* Mart. K (FsK) growth

The fungal strain *F. solani* Mart. FsK is an endophyte, originally isolated from tomato roots (Kavroulakis et al. 2007). The fungus was cultivated on autoclaved (121 °C for 20 min) potato-dextrose-agar medium (PDA) supplemented with total extracts of non-treated *in vivo* plant and non-treated *in vitro* nodal segments. From each sample, the amount of extract used in the PDA culture medium was determined based on the concentration of 1 mg/L of RA. The extracts were solved in 50:50 (V:V) methanol/water and 10 μL of the filtered solvent were added to the PDA media. The media without any solvent was considered as a control. An agar plug of FSK was placed in the middle of a petri dish and incubated in growth chambers in the dark at 25 °C. The number of conidia, fresh weight and dry weight of FsK were measured under treatment of control, *in vitro* and *in vivo* extracts of *S. khzistanica* plants. On the other hand, callus induction of *S. khzistanica* was performed following the method which was done by Ghorbanpour and Hadian (2015). In order to study the effect of calluses on FsK growth, two callus pieces were placed on two sides of each petri dish containing fungi. The inhibitory effects of these calluses on FsK growth was assayed by measuring the diameter growth of FsK every day for 6 days.

### Statistical analysis

All the experiments were set up in completely randomised design (CRD) with three replications. The data were subjected to analysis of variance (ANOVA) using SPSS software (version 18.0). The means of treatments in each experiment were separated at *P* = 0.05 level of significance according to Duncan’s multiple range test (DMRT).

## Results

### Establishment of nodal segments culture in liquid medium

**Figure 1.**
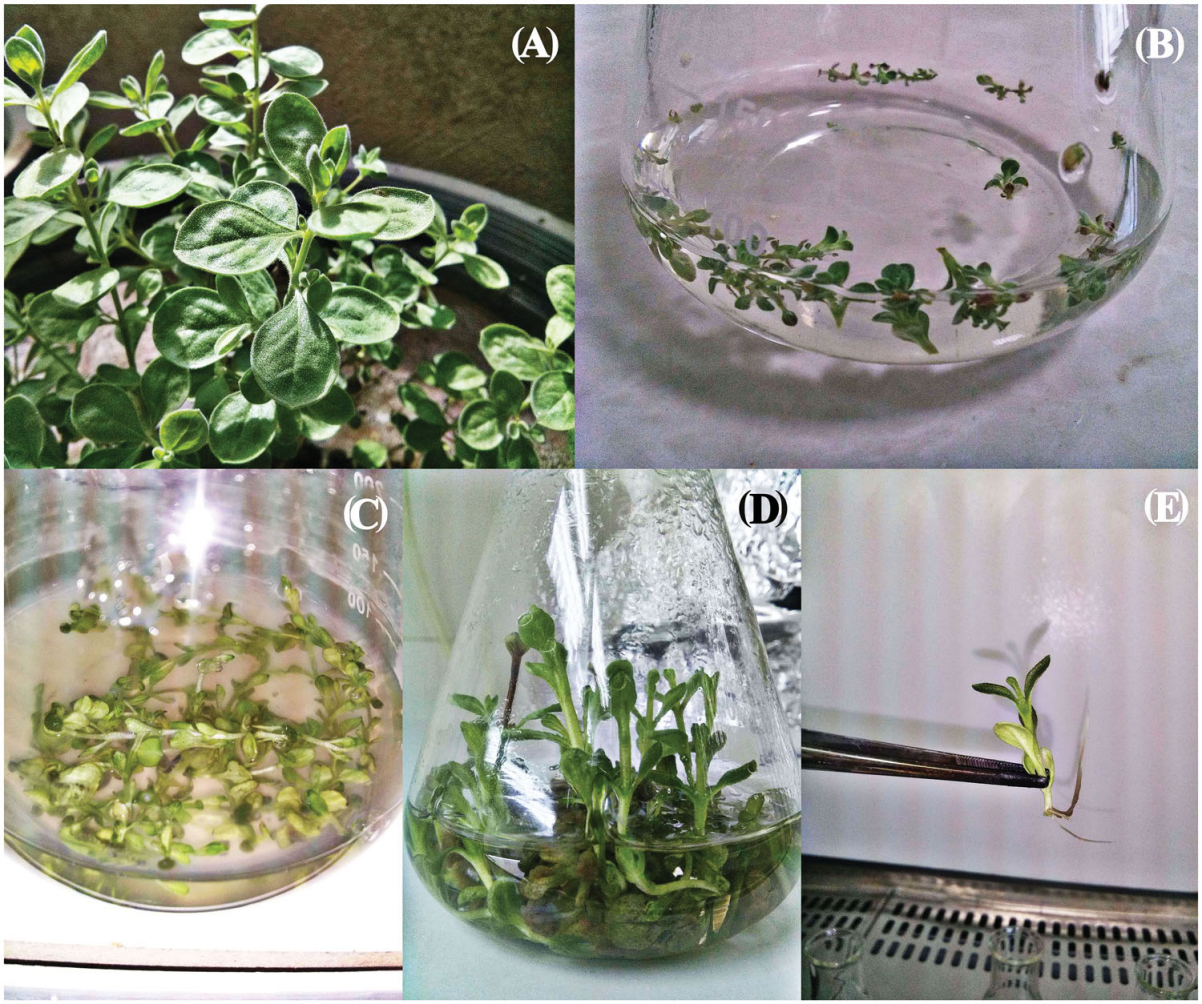
Plant regeneration from *Satureja khuzistanica* nodal segments in liquid MS culture medium. (A) *Satureja khuzistanica* plant at reproductive stage used to prepare nodal segment explants, (B) Culture initiation time after sterilising nodal segments suspended in 100 mL MS culture medium, (C) One-weak-old samples after initiation time, (D) Two-weak-old samples and the time for sub culturing, of nod explants, (E) Root induction in some regenerated plantlet from nodal segments cultures.

As shown in Figure 1(A–E), the suitable nodal segments (2 cm in size) derived from green house plants (Figure 1(A)), after culture in liquid MS culture medium (Figure 1(B)), were quickly regenerated to whole plantlets after 2 weeks from culture initiation (Figure 1(D)). Some of regenerated plantlets were rooted in this culture condition (Figure 1(E)).

### RA quantification under *in vitro* condition

**Figure 2.**
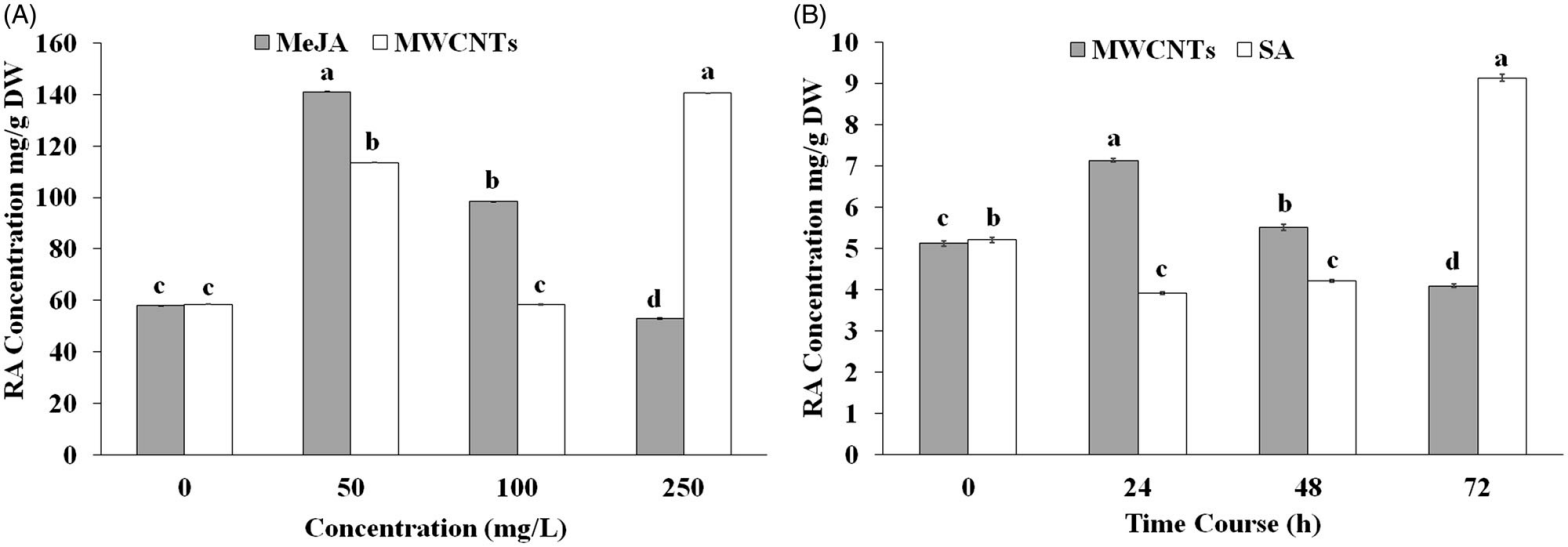
Contents of RA in *Satureja khuzistanica* plants derived under *in vitro* and *in vivo* conditions. (A) Content of rosmarinic acid in nodal segments cultures of *S. khuzistanica* treated with different concentrations of MeJA and MWCNTs. (B) Content of rosmarinic acid in *in vivo* plants treated with 100 mg/L MWCNTs and 200 mg/L SA at different time points. The levels of rosmarinic acid in each sample were analysed by HPLC. Different letters indicate significant differences (Duncan’s multiple range test, *p* *<* 0.05). Bars represent means ± SD.

In this experiment, the RA content was assessed in a nodal segment culture of *S. khuzistanica* under MeJA and MWCNT elicitors at 0, 50, 100 and 250 mg/L. Treated cultures with different concentrations of both MeJA and MWCNTs elicitors showed significant differences (*p* < 0.05) in RA content (Figure 2(A)). The amount of RA was increased under both treatments in nodal segments culture of *S. khuzistanica.* The highest amount of RA was obtained in nodal segments culture treated with 50 mg/L MeJA (140.99 mg/g DW, Figure 2(A)) and 250 mg/L MWCNTs (140.49 mg/g DW, Figure 2(A)), which was 2.43-fold higher than untreated cultures (57.87 mg/g DW). RA accumulation was enhanced by the addition of 50 mg/L of MWCNTs and MeJA, and then it noticeably decreased with 100 mg/L of both treatments (Figure 2(A)). MWCNTs at 50 and 250 mg/L, raised RA contents to 113.54 and 140.49 mg/g DW, which was 1.96‐ and 2.42-fold higher than the content in the control, respectively, while the amounts of RA in nodal segments culture treated with 50 and 100 mg/L MeJA were higher than control (140.99 and 98.31 mg/L DW, respectively, Figure 2(A)).

### RA quantification under *in vivo* condition

In this experiment, the RA content was investigated in *in vivo* derived shoots of *S. khuzistanica* under both SA and MWCNTSs elicitors over a period of time from 0 to 72 h. The quantification of RA was performed by using the standard calibration curve method. *S. khuzistanica* plants treated with 100 mg/L MWCNTs and 200 mg/L SA showed significant differences (*p* < .05) in concentrations of RA at different time points after both treatments (Figure 2(B)). The results demonstrated that the highest amount of RA was noticed at the time point of 24 h after the MWCNTSs treatment and 72 h after SA treatment (7.13 mg/g DW and 9.12 mg/g DW, respectively) which was 1.5- and 1.8-fold higher than untreated plants, respectively (Figure 2(B)). The RA content was increased under MWCNTSs elicitor, 24 h after treatment, while the reduction was observed at 48 h and reached to less than control after 72 h (Figure 2(B)). RA production was decreased 24 and 48 h after SA treatment (2.91 and 1.89 mg/g DW, respectively) while a dramatic increase was observed after 72 h from initial treatment (Figure 2(B)).

### Changes of antioxidative enzymes under MWCNTSs and MeJA treatments *in vitro*

**Figure 3.**
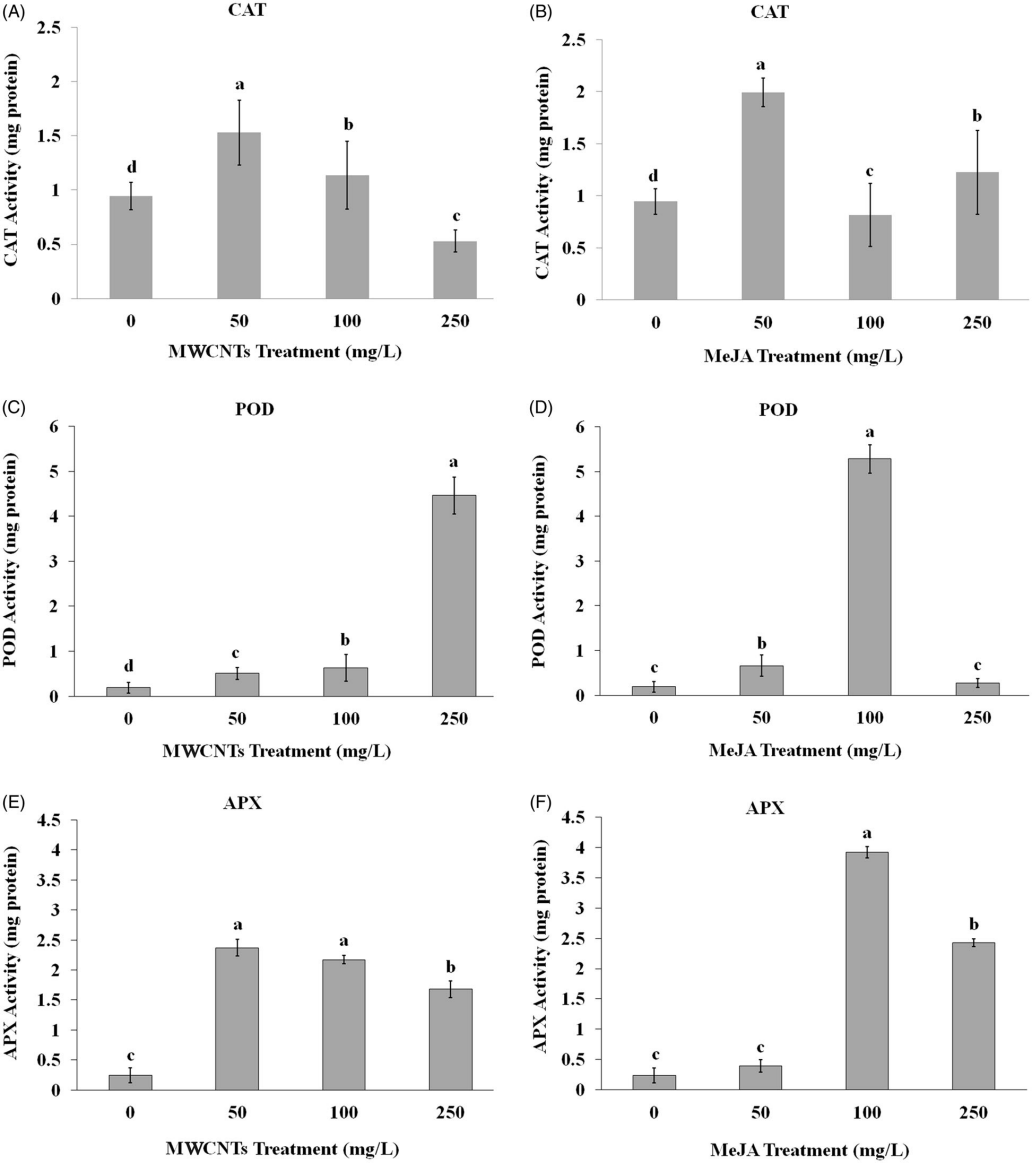
Effects of different concentrations of MWCNTSs and MeJA treatments on catalase (A, B), guaiacol peroxidase (C, D) and ascorbate peroxidase (E, F) activities in *Satureja khuzistanica* nodal segments cultures. Different letters indicate significant differences (Duncan’s multiple range test, *p <* 0.05). Bars represent means ± SD.

The response of CAT, POD, and APX enzymes were variable at different concentrations of MWCNTSs and MeJA treatments (Figure 3(A–F)). The maximum activity of CAT enzyme was obtained with 50 mg/L of MWCNTSs and gradually reduced by application of 100 and 250 mg/L of MWCNTSs treatments (Figure 3(A)). Also, the highest CAT activity was observed when 50 mg/L MeJA was used in nodal segments culture medium (2 mg protein, Figure 3(B)). POD activity was significantly increased at 250 mg/L MWCNTSs compared to control and other MWCNTSs treatments (Figure 3(C)), while use of 100 mg/L MeJA in culture medium significantly enhanced the activity of this enzyme up to 28.23-fold (5 mg protein) compared to control and other MeJA treatments (Figure 3(D)). Application of 50 and 100 mg/L MWCNTSs in nodal segments culture medium significantly enhanced the activity of APX enzyme compared to control, while APX activity was decreased at 250 mg/L MWCNTSs (Figure 3(E)). With 100 mg/L MeJA in culture medium, APX activity was significantly enhanced (up to 16.33-fold, 4 mg protein) whereas its activity reduced around −1.5-fold at 250 mg/L MeJA (Figure 3(F)).

### Changes of antioxidative enzymes under MWCNTSs and SA treatments *in vivo*

**Figure 4.**
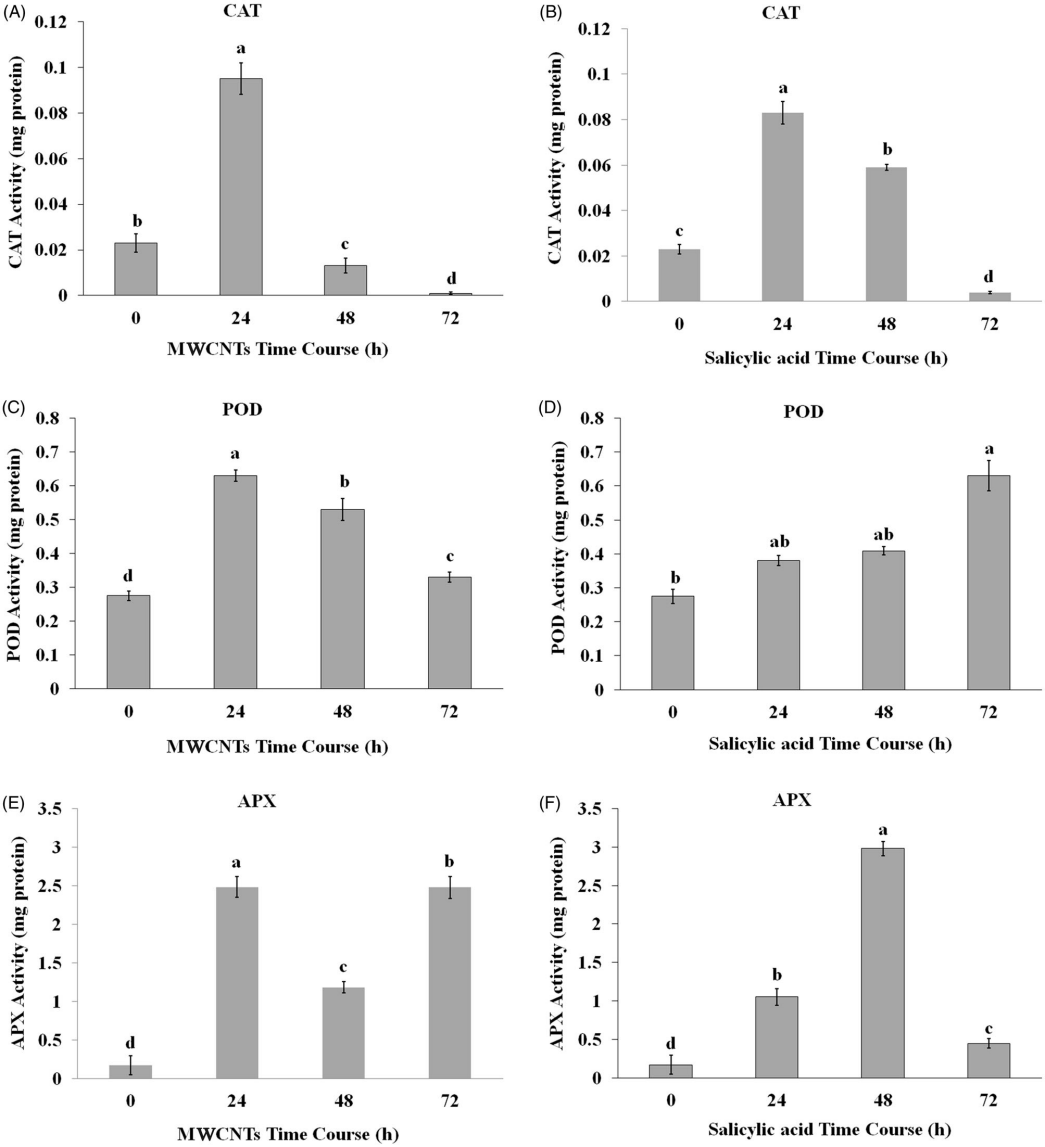
Enzyme activities of *Satureja khuzistanica* plants treated with 100 mg/L MWCNTs and 200 mg/L SA at 24, 48, 72 h after initial treatments. (A, B) catalase activity, (C, D) guaiacol peroxidase activity, (E, F) ascorbate peroxidase activity. Different letters indicate significant differences (Duncan’s multiple range test, *p* *<* 0.05). Bars represent means ± SD.

In this experiment, significant differences (*p* < 0.05) were observed in CAT, POD and APX enzyme activities in *S. khuzistanica* plants treated with 100 mg/L MWCNTs and 200 mg/L SA at different time points after treatment. CAT activity was significantly increased under both MWCNTSs and SA treatments 24 h after eliciting, while it was gradually decreased 48 and 72 h after initial elicitation (Figure 4(A,B)). POD activity was significantly enhanced in plant extracts treated with either 100 mg/L MWCNTs or 200 mg/L SA, 24 h after initial treatment, but the activity of this enzyme was gradually decreased 48 and 72 h after MWCNTs treatments (Figure 4(C)) while it was increased 72 h after SA treatment (Figure 4(D)). The APX activity does not follow a specific pattern, was raised after 24 h under both MWCNTs and SA treatments (Figure 4(E,F)), whereas 48 h after initial elicitation, its activity was reduced under MWCNTs treatment (Figure 4(E)) and enhanced by SA treatment (Figure 4(F)). The APX activity showed a significant increase, 72 h after MWCNTs treatment (Figure 4(E)) while its activity was reduced 72 h after SA treatment (Figure 4(F)).

### Antioxidant activity under MWCNTSs and SA treatments *in vivo*

**Figure 5.**
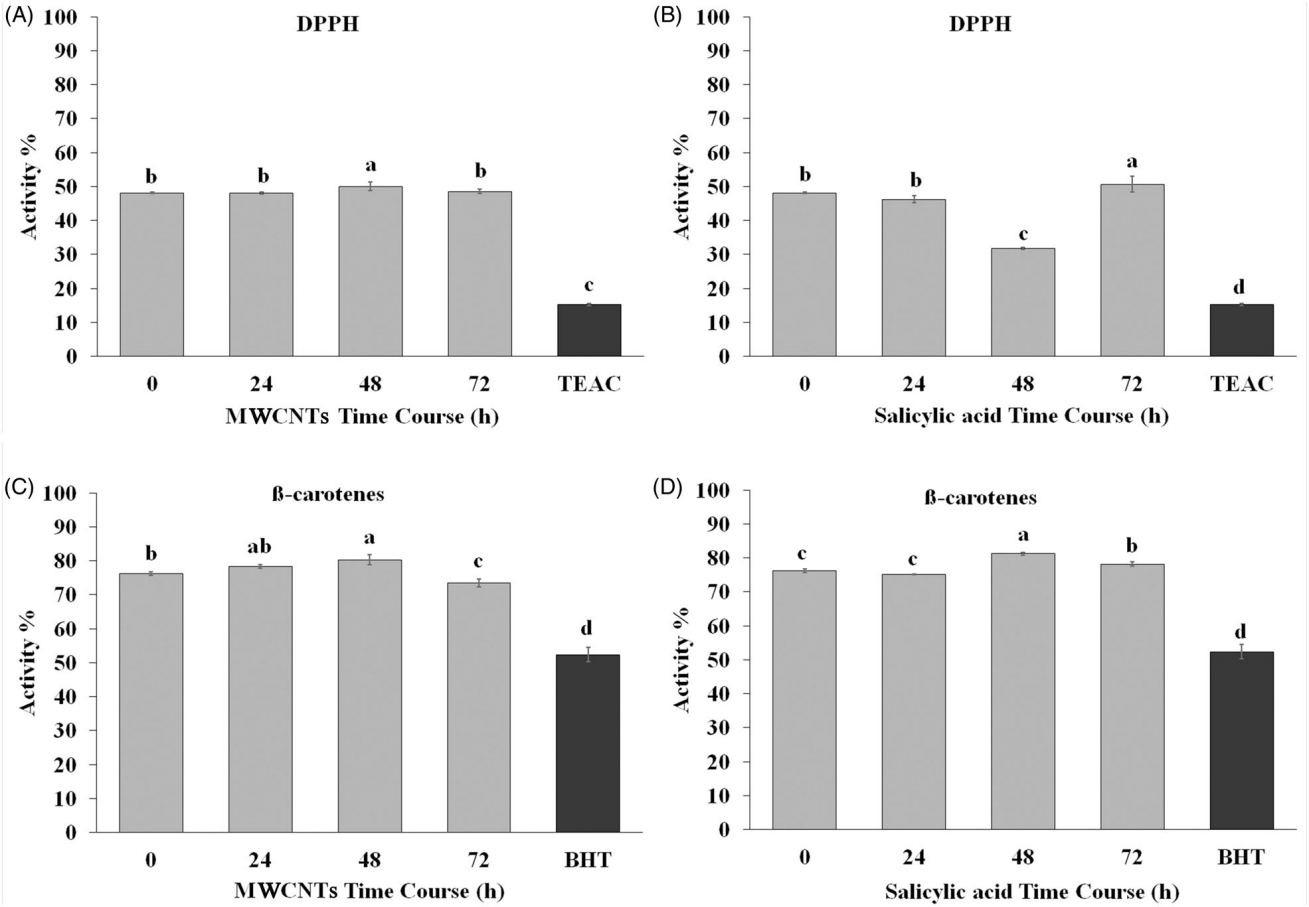
Antioxidative properties of *Satureja khuzistanica* extracts under MWCNTs and SA treatments. (A, B) DPPH assay (%) in *Satureja khuzistanica* extracts under MWCNTs and SA treatments at different time points after initial treatment, (C, D) β-carotene bleaching tests (%) in *Satureja khuzistanica* extracts under MWCNTs and SA treatments at different time points after initial treatment. Different letters indicate significant differences (Duncan’s multiple range test, *p* *<* 0.05). Bars represent means ± SD.

The free radical scavenging activity of the elicited samples was evaluated using DPPH and β-carotene leaching assays. The results indicated that the extracts obtained from *in vivo* plants treated with 100 mg/L MWCNTs and 200 mg/L SA exhibited a significant induction of antioxidant activity. It seems that *S. khuzistanica* extracts possessed strong radical scavenging activity in comparison to a standard antioxidant trolox. It was also found that *S. khuzistanica* hydromethanolic extract caused around 80% inhibition in formation of peroxidation products in comparing to its standard BHT (52.37%) in β-carotene bleaching test (Figure 5). Antioxidant activities from all MWCNTs and SA treated samples at different time points were significantly stronger than that of positive control reagent (TEAC) in DPPH assay (50% inhibition, Figure 5(A,B)). The complementary measurement of antioxidant activity was done using β-carotene leaching test, and the similar tendency was found as DPPH assay (Figure 5(C,D)). The highest antioxidant activity in DDPH assay was observed in *S. khuzistanica* extracts 48 h after MWCNTs treatment (Figure 5(A)) and 72 h after SA treatment (Figure 5(B)). In β-carotene bleaching test, the highest values of antioxidant activity were obtained after 48 h from both MWCNTs and SA treatments (Figure 5(C,D)).

### The effects of *in vivo* and *in vitro* extracts on FsK growth

**Figure 6.**
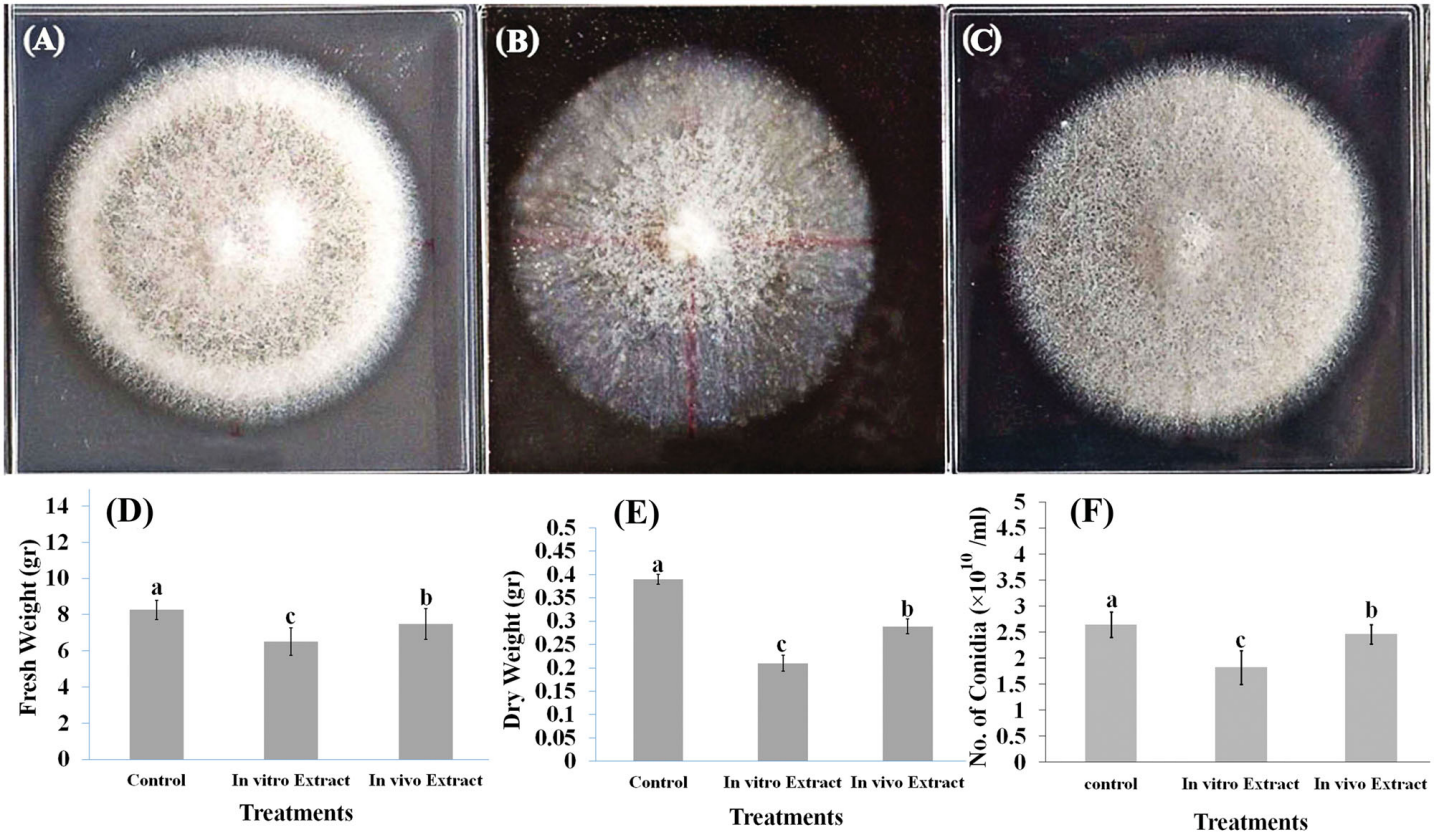
The effect of *Satureja khuzistanica* plant extracts containing 1 mg/L rosmarinic acid on FSK aggregation, fresh weight, dry weight and number of conidia. (A) PDA culture medium without *S. khuzistanica* extracts, (B) PDA culture medium containing the non-treated *in vitro* nodal segments extract, (C) PDA culture medium containing the non-treated *in vivo* plant extract, (D) Effect of control, *in vitro* and *in vivo* extracts of *Satureja khuzistanica* plants on fresh weight of FSK, (E) Effect of control, *in vitro* and *in vivo* extracts of *Satureja khuzistanica* plants on dry weight of FSK, (F) Effect of control, *in vitro* and *in vivo* extracts of *Satureja khuzistanica* plants on the number of conidia of FSK. Different letters indicate significant differences (Duncan’s multiple range test, *p* *<* 0.05). Bars represent means ± SD.

**Figure 7.**
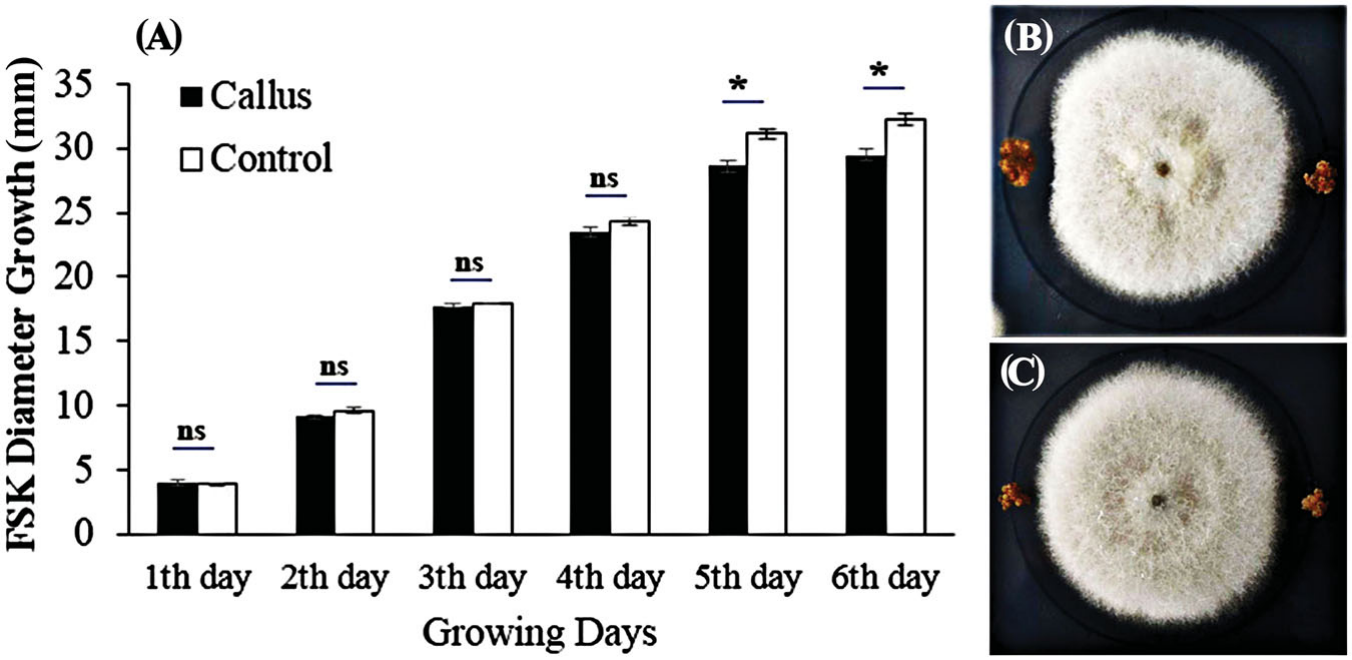
The inhibitory effect of *Satureja khuzistanica* calluses on FSK growth. (A) The effects of callus on FSK diameter growth during 6 days. At above ns (*p* > 0.05), *(*p* < 0.05), shows the result of separate analysis of variance at each day between control and callus treatment. Bars represent means ± SD, (B, C) Decrease the growth of FsK in the vicinity of the *Satureja khuzistanica* callus.

Non-treated *in vivo* and *in vitro* (nodal segments) extracts and callus derived from *S. khuzistanica* were used in order to assess their ability to inhibit FsK growth. In an attempt to evaluate the impact of extracts and callus, FsK growth was significantly affected in all cases. As shown in Figure 6, the aggregation of the control samples (Figure 6(A)) was higher than both *in vivo* and *in vitro* samples. *In vitro* nodal segments extract (Figure 6(B)) had a more inhibitory effect on FsK growth aggregation than *in vivo* (Figure 6(C)) one. The FsK aggregation was the lowest for *in vitro* extract in fresh weight (6.51 g, Figure 6(D)), dry weight (0.21 g, Figure 6(E)) and number of conidia (1.82 × 1010, Figure 6(F)), The results showed that the callus does not have any effect on the growth rate of FsK till the end of the 4th day, but significant result was observed on 5th day (2.75 mm, Figure 7(A–C)).

## Discussion

*In vitro* techniques can accelerate biosynthesis of secondary metabolites. In some cases, such as *S. khuzistanica*, *in vivo* culture could not be easily developed due to the seed dormancy and very low germination rate (Gupta et al. 2011). In the present study, we found that nodal segments culture of *S. khuzistanica* in liquid medium could dramatically enhance the RA content in this plant species and was considered as a new source of RA. Therefore, these *in vitro* cultures concomitant with green house derived plants of *S. khuzistanica* were used as plant materials for this study. In the first part of this study, we found that the RA contents were positively influenced by different concentrations of MeJA and MWCNTs in a nodal segment culture of *S. khuzistanica*. In accordance with these results, MeJA and MWCNTs treatments significantly increased the amount of RA in cell suspension cultures of *Coleus blumei* Benth. (Lamiaceae) (Szabo et al. 1999) and callus cultures of *S. khuzistanica* (Ghorbanpour and Hadian 2015), respectively. It was supposed that the high amount of RA observed 48 h after treatments, as optimum time for *in vitro* culture of Lamiaceae plants (Krzyzanowska et al. 2011; Park et al. 2016).

It was reported that the plant phytohormones such as MeJA, as a main compound in signal transduction pathway, had an important role to induce plant defence against subsequent attack of stresses (Bulgakov et al. 2002) through various biochemical, physiological and molecular mechanisms (Hyun-Jin et al. 2007). Application of exogenous MeJA caused to produce high levels of secondary metabolites in various plants (Hyun-Jin et al. 2007). Also, carbon nanomaterials, had been found to apply in plants due to their unique characteristics and could have great impacts on plant secondary metabolite. However, it has been suggested that high doses of MWCNTs have cytotoxicity effects on plant cells due to their density (Lei et al. 2008; Hadian et al. 2011; Vicent and Plasencia 2011; Khodakovskaya et al. 2012; Villagarcia et al. 2012; Zhang et al. 2013; Hatami and Ghorbanpour 2014; Ghorbanpour and Hadian 2015). As a result, it can cause structural damage in plant cells and conditional death.

According to this result, in the present study, decreasing the RA content from 50 to 100 mg/L might be due to toxicity of MWCNTs, while increasing it from 100 to 250 mg/L could be related to activation of defensive system in plants (Ghorbanpour and Hadian 2015). Translocation studies of carbon nanotubes assumed that an interaction happened between cell wall and carbon nanotubes through adherence forces such as hydrogen bonding (Dapkevicius et al. 1998). Thus, a penetration could be taking place through the apoplastic pathway and envelop to the surface of cells till arriving at a narrower passage than their size, being then accumulated and leading to change in metabolic functions as well (Villagarcia et al. 2012). There is a lack of knowledge regarding the effects of MWCNTs on plant growth and metabolic features. However, results from this study suggested that the metabolic characteristics were markedly influenced by the employed both MWCNTs and MeJA.

In the second part of this study, the RA contents were evaluated in greenhouse grown plants treated with 100 mg/L MWCNTs and 200 mg/L SA. The quantitative HPLC analysis showed the higher amount of RA in treated plants rather than untreated one. The highest content of RA was obtained at the time point of 72 h after SA treatment. Also, MWCNTSs treatment at 24 h could be supposed as threshold time. Phytotoxicity of MWCNTs at the time points of 48 h and 72 h may have led to low RA content. It has been suggested that application of excess SA, an endogenous growth regulator, causes to the uptake of exogenous SA into the cells that result in H_2_O_2_ accumulation and oxidative defence. Maffei et al. (2007) reported that H_2_O_2_ could increase the production of secondary metabolite in plants. SA generated a wide range of metabolic and physiological responses which were involved in plant defence. It also activated the ROS generation and other defensive procedures that could help the plant cells to withstand oxidative damage, specifically to cope with ROS generated by the oxidative stress (Ghorbanpour and Hadian 2015). Our results were in accordance with the reports where exogenous application of SA induced H_2_O_2_ content and oxidative enzymes in *S. khuzistanica* to overcome oxidative stress.

### Changes of antioxidative enzymes under MWCNTSs and MeJA treatments *in vitro*

The next part of this study aimed to evaluate the ability of exogenously applied MeJA and MWCNTs to *S. khuzistanica* nodal segments culture, to overcome stress-induced responses involving antioxidative enzymes and ROS. The activities of CAT, POD, and APX enzymes were estimated under the mentioned treatments. The responses of given enzyme were variable at different concentrations of MWCNTSs and MeJA treatments. ROS are cytotoxic, have important roles in cell signalling and homeostasis, regulating the expression of genes related to antioxidant defence mechanisms (Neill et al. 2002). Plants have a highly efficient protective enzyme, including antioxidant enzyme defence system, POD, APX, and CAT to scavenge ROS, enhancing plant tolerance to different stress factors (Jiang and Zhang 2002). Nano materials could cause higher enzyme loading due to their higher surface area (Kim et al. 2008). An augmentation in CAT, POD, and APX activities at different concentrations of MWCNTs treatments showed the ability of MWCNTs to induce plant defence system. It had been shown that applications of MWCNTs could increase the activities of antioxidant enzyme POD (Ghorbanpour and Hadian 2015). It can be assumed that enhance the peroxidase activity is related to the oxidative stress caused by MWCNTs.

Our results demonstrated that the level of enzyme activity decreases by enhancing the MWCNTs concentration that could be due to inactivation of related molecules by MWCNTs or other chemical interactions. This result was compatible with the result of Smirnova et al. (2011). They showed that peroxidase activity was enhanced in *Onobrychis arenaria* (Kit.) DC. (Leguminosae) seedlings at the concentrations of 100 and 1,000 mg/L MWCNTs, which was significantly higher than the control (Smirnova et al. 2011). The same results happened to CAT and APX enzymes in this study. It was shown that increase in the activities of antioxidant enzymes is caused by exogenous application of MeJA (Anjum et al. 2011), leading to oxidative stress in plant cells. The antioxidant enzymes can play protective roles against the harmful effects of ROS, which is observed at initial moments of MeJA exposure (Afkar et al. 2013). The plants use enzymatic-antioxidant systems to minimise the damage caused by ROS (Smirnova et al. 2011). In our study the enhancement of CAT, POD and APX enzyme activities can be due to stimulation of the defence system against ROS, while the decrease in the enzyme levels may be caused by hyper toxicity of ROS. As reported by Woodward and Bartel (2005), the increase of peroxidase enzyme activity changes the plant stress hormone levels resulting in oxidative defence in plants. In accordance with the results of this study, the exogenous treatment of *Mentha piperita* L. (Lamiaceae) with MeJA significantly increased the activity of antioxidant enzymes (POD, SOD) (Afkar et al. 2013).

### Changes of antioxidative enzymes under MWCNTSs and SA treatments *in vivo*

Relative to the control of this experiment, the MWCNTs at the time point of 72 h suppressed the CAT activity in *S. khuzistanica*, which were related to the high ROS content and intense oxidative damage. Previous reports have also confirmed that the application of carbon nanomaterials caused an increase in ROS generation, which subsequently induced different detrimental effects (Dapkevicius et al. 1998; Ghorbanpour and Hadian 2015). According to Khodakovskaya et al. (2012) carbon nanomaterials could up-regulate the genes which were involved in stress signalling and molecular response as biotic elicitors do. The highest CAT activity ratio in plants exposed to the MWCNTs and SA treatments at the 24 h time point indicated the maximum activity of CAT enzyme and oxidative defence. Here, up-regulation of RA biosynthesis following exposure to 100 mg/L MWCNTs at the time point of 24 h was linked to the highest CAT, POD and APX activities. In contrast, a greater elevation in amount of RA was found in plants treated with SA at spraying time point of 72 h, which a rise in POD activity has been shown, might refer to the crucial role of POD in RA biosynthesis and oxidative defence. No significant differences were recorded in POD activities between 24 h and 48 h treated plants. The sudden decrease in CAT activity from 24 h to 72 h and reduction of APX activity at 72 h could be pointed to the phytotoxicity of plant at both treatments.

### Antioxidant activity

The plant flavonoids and phenolic content are important phytochemicals that possess free radical scavenging activities mainly because of their redox properties (Duarte-Almeida et al. 2006). The results indicated that *S. khuzistanica* extracts, treated with SA and MWCNTSs, caused around 80% inhibition in the formation of peroxidation products compared to its standard BHT in β-carotene bleaching test and TEAC standard for DPPH assay (50% inhibition). Also, the antioxidant activity of RA has been proven previously through both *in vitro* (Chen and Chen 1999) and *in vivo* experiments. These results suggest that the SA (Bulgakov et al. 2002; Vicent and Plasencia 2011; Xu et al. 2011) and MWCNTSs can play a vital role in the production of phytomedicine by stimulating plant *in vivo*, which would be very beneficial in understanding the fundamental molecular mechanisms of metabolites elicitation.

### Inhibitory effects of *in vivo*, *in vitro* and callus on FsK growth

The extracts and callus treatments demonstrated successful inhibitory impacts on the growth rate of FsK in comparison to the control. We hypothesised that reduction in FsK growth may be related to the RA inhibitory effect which was produced in plants and activated resistance mechanisms in natural condition. The same results observed in tomato plants (Garantonakis et al. 2018) confirmed our understanding. It is clear that all the extracts and callus showed an inhibitory effect on FsK aggregation (Figure 6) and diameter growth (Figure 7). The lowest FsK aggregation for *in vitro* extract in number of conidia (1.82 × 1010), fresh weight (6.51 g) and dry weight (0.21 g) and on the other hand, the reduction in FsK growth diameter to 2.75 mm on 5th in callus treatments demonstrated the inhibitory effects of RA on FsK growth. These results suggested that the growth diameter and aggregation of FsK was inhibited by the callus and RA induction compared to the control. Bais et al. (2002) showed that fungal elicitors induced a rapid increase in RA production and RA was over-produced to inhibit fungal growth due to its polyphenolic properties.

## Conclusions

The present study demonstrated that MeJA, SA and MWCNTSs induced different biochemical and physiological processes with different responses both *in vivo* and *in vitro*. They presumably elevated the level of oxidative stress in the *Satureja* plants through inducing an up-regulation in the gene expression level involved in the flavonoid metabolism resulting to activate specific key enzymes such as CAT, POD and APX. These results could be utilised for the induction of plant defensive system against ROS that will enable the plant to withstand many biotic and abiotic stresses. However, it caused a decrease in the RA contents and enzyme activity in severe oxidative stress. These results draw attention to the fact that exogenous application of MeJA, SA, and MWCNTSs influences biochemical and physiological aspects of *S. khuzistanica* plant. They can act as an effective elicitor to enhance the biosynthesis of pharmaceutically active molecules such as RA, which may be considered as part of the daily human diet in treatment of several diseases.
